# Recovery-oriented care in Teams Working with the ART Model in long-term Mental Health care: A Qualitative Study on the Experiences of Service Users and Their Significant Others

**DOI:** 10.1007/s10597-024-01269-4

**Published:** 2024-04-15

**Authors:** Lieke Zomer, Lisette van der Meer, Jaap van Weeghel, Guy Widdershoven, Isa de Jong, Yolande Voskes

**Affiliations:** 1 Department of Ethics, Law and Humanities, Amsterdam UMC, Location VUmc, Amsterdam, the Netherlands; 2 Department of Clinical & Developmental Neuropsychology, University of Groningen, Groningen, the Netherlands; 3 Department of Rehabilitation, Lentis Psychiatric Institute, Zuidlaren, the Netherlands; 4 Tranzo Scientific Center for Care and Wellbeing, Tilburg University, Tilburg, the Netherlands; 5 Impact Care Group, GGz Breburg, Tilburg, the Netherlands; 6 Altrecht GGz, Zeist, the Netherlands

**Keywords:** Recovery-oriented care, ART model, Service user perspective, Significant others, Qualitative research

## Abstract

The Active Recovery Triad (ART) model provides a framework for recovery-oriented care in long-term mental health settings. The aim of this study is to gain insight into the experiences and views of service users and their significant others regarding care and support they receive from teams working with the ART model. Semi-structured interviews were performed with nineteen service users and five significant others of teams operating in Dutch long-term mental health care. Data were analyzed using thematic analysis. The three core principles of the ART model formed the deductive basis of the analysis and for every principle themes were identified inductively. Under the principle ‘Active’, service users mentioned that they feel motivated, work actively on personal recovery goals and have dreams for the future. Service users valued the service as a safe environment, but several service users also preferred to live more independently. Under the principle ‘Recovery’, participants reported how the dimensions of recovery (health, personal identity, daily life and community functioning) were addressed in care and support. Also, specific conditions for recovery-oriented care were identified, for example regarding specific expertise of care providers. Under the principle ‘Triad’ the support from significant others, contact with care workers and with other service users were identified as important. The insights regarding what is important for service users and their significant others may contribute to the improvement of care and support in long-term mental health care. In addition, the findings of this study provide directions for the further development of the ART model.

## Introduction

In many countries, the deinstitutionalization process played an important role in the reform of the mental health sector the past decades, including reducing the number of beds in large hospitals and providing care and support preferably in service users’ own home (Tsai & Salyers, 2010; van der Meer & Wunderink, 2019). A central aspect in this reform was the emergence of the concept of recovery and the provision of recovery-oriented care, which provided a new and hopeful perspective to support service users to live a meaningful life, despite the limitations of their illness (Anthony, 1993). However, these developments were challenging for long-term facilities (De Ruysscher et al., 2020; Waldemar et al., 2016). Service users in long-term setting are people with complex needs due to a serious mental illness and therefore were considered as being dependent on 24-hour care and support for a longer period of time. This group of people was often regarded as permanent residents of these facilities. Yet, based on views of the recovery movement, a better perspective for this group of people should be possible. This was also supported by literature following the deinstitutionalization process in other countries, such as the UK (Taylor Salisbury et al., 2017; Trieman & Leff, 2002). Through focusing on chances and opportunities in the recovery process, after years of focusing merely on stabilization and reduction of symptoms, steps in the recovery process may be possible for this group of service users as well.

The Active Recovery Triad (ART) model provides a framework for recovery-oriented care in long-term mental health settings (van Mierlo et al., 2016; Zomer et al., 2020). The starting point of care and support according to the ART model are the needs, strengths and wishes of service users and their significant others, in order to work on personal recovery goals. The three core principles of the ART model are Active, Recovery and Triad. First, “Active” refers to the active attitude that is required of all parties involved, including service users, their significant others and care workers. It also involves active listening to the preferences of service users regarding their living situation and regular critical evaluation of the provided care and support. Second, “Recovery” includes different dimensions of recovery that should be taken into account in care for and support of service users. In the ART model, four dimensions are distinguished, as commonly used in Dutch mental health practice, namely recovery of health (mental health, physical health and the intertwinement between those two), recovery of identity (exploring someone’s life story and (re)discovering their identity), recovery of daily functioning (including (re)gaining grip on daily tasks to become more self-reliant), and recovery of community functioning (participation and having a social role in the community) (Dröes & Plooy, 2010; Swildens et al., 2018). Third, “Triad” refers to the collaboration between care workers, service users and their significant others, working with (family) peer workers that represent these perspectives in the team, and involving service users and significant others in team and organization policy.

The ART model provides guidelines for recovery-oriented care in this long-term psychiatric practice. This includes for example the use of specific recovery-oriented interventions, education for staff, collaboration to foster community participation and tools to stimulate the collaboration in the triad. It also provides suggestions for improvements of the care process, including care coordination meetings, working with a personal recovery plan and structural evaluation of recovery goals in the triad. The model also emphasizes the importance of the culture and vision of the team and suggests a shared recovery-oriented vision, a recovery-oriented attitude of staff and a close collaboration between different disciplines involved. Finally, the model provides suggestions for conditions that the organization of care should meet, such as a healing environment, the facilities for service users and the different disciplines in the team (Zomer et al., 2022).

The ART model was introduced in the Netherlands in 2016, based on an iterative development process with many stakeholders, including the perspectives of (ex-)service users, significant others, care workers, researchers, managers and directors from the field of long-term mental health care. To date, a large number of mental health care organizations (> 20) in the Netherlands have started implementing the ART model in practice. In another study we investigated the association between the degree of compliance to the ART model and the recovery of service users using quantitative questionnaires, in order to provide insight into more general patterns. We aim to publish the results of this study elsewhere. Among others, we found that elements of the ART model were associated with outcomes regarding recovery of service users and satisfaction regarding care and support, as measured with various quantitative instruments. However, little is known of the personal experiences of service users and their significant others regarding care and support by teams working with the ART model. Therefore, the aim of the current study is to investigate the experiences and views of service users and their significant others regarding care and support by teams working with the ART model. We will focus on three questions related to the core principles of the ART model, namely Active, Recovery and Triad: (1) How do service users and significant others experience the Active principle of the model, aiming at motivation and active involvement in care and support? (2) What are the experiences of service users and significant others regarding the Recovery principle of the model, more specifically the four dimensions of recovery? (3) How do service users and significant others experience the Triad principle, including collaboration with all parties involved? We will investigate these questions using a qualitative approach.

## Methods

### Study Design

In order to study experiences and views of service users and significant others, a qualitative approach is recommended to get at the heart of these personal processes (Stanhope & Solomon, 2008). We performed semi-structured interviews with service users and significant others of teams operating in long-term mental health care and in the process of ART implementation. We used the Standards for Reporting Qualitative Research (SRQR) in reporting the current study (O’Brien et al., 2014).

### Recruitment of Participants

A purposive sampling approach was performed in the recruitment of participants (Thorogood & Green, 2018). We recruited service users and significant others from teams that were in the process of implementing the ART model. Eighteen teams participated in the quantitative study on the association between the ART model, recovery-oriented care and recovery of service users. The results of this study will be published elsewhere. The data collection of the current study was performed simultaneously to the follow-up measurement of this quantitative study. We asked the teams to invite at least one of their service users to participate in this study. Preferably a family member or significant other of this service user was also invited to participate. Service users of these teams were people with serious mental illnesses and, because of their mental health needs, considered as dependent on 24-hour care and support in a long-stay clinical ward or sheltered housing facility. Although there is a large variety of settings for long-term, 24-hour care and support (McPherson et al., 2018), in the Dutch context a distinction can be made between clinical wards and sheltered housing facilities. Long-stay clinical wards are open or closed wards, often situated at large institutional grounds. At these wards both treatment and 24-hour support are provided to service users by multidisciplinary teams including staff providing treatment as well as daily and practical support. Sheltered housing facilities are often situated in the community and include teams that provide support to foster self-reliance and community participation. These teams cooperate with outpatient psychiatric care teams when psychiatric treatment is needed. People were eligible to participate if they (or their significant others) were in care at the current location for a minimum of three months and if they were able to concentrate for the interview.

### Data Collection

Prior to the interviews, a topic list was developed (see supplementary information). The three central research questions of this study, related to the three core principles of the ART model (Active, Recovery and Triad), provided the basis of this topic list. Interview questions focused on the experiences and views of the participants regarding care and support according to these three core principles, for example what participants’ experiences were regarding the active attitude of care workers, their significant others and themselves, questions regarding different dimensions of recovery and questions regarding the collaboration in the triad. In addition, questions regarding the participants’ opinion on success factors and points for improvement of care and support were included. The interviews took place in the period between January and June 2022. The main researcher (LZ) performed the interviews at the location (ward/housing facility) of the participants. The duration of the interviews was between 30 and 60 min and they were audio recorded for analysis.

### Data Analysis

A thematic analysis was performed to analyze the data (Thorogood & Green, 2018). The three research questions, reflecting the three core principles of the ART model, formed the deductive basis of the analysis. For every research question we developed (sub)themes based on the data. Therefore, a combination of inductive and deductive approach was used (Fereday & Muir-Cochrane, 2006). The transcripts of the audio recordings were read and coded by two researchers (LZ and IdJ) using MAXQDA version 2020 (VERBI Software GmbH, 2020). Codes were discussed among the two researchers to check whether similar codes were identified based on the transcripts (triangulation). If not, these differences were discussed until consensus was reached. Subsequently, for each research question, the codes were clustered into themes and subthemes. The (sub)themes were discussed among the three researchers (LZ, IdJ and YV) until consensus was reached upon the content, distinctiveness and relevance. Suitable titles were developed for each of the (sub)themes and discussed among the involved researchers. The final themes were discussed among all authors.

### Researcher Characteristics and Reflexivity

The research team includes experienced qualitative (and quantitative) researchers. All authors have an interest in research related to recovery, rehabilitation, participation and the reduction of coercion in psychiatry. Three authors (LvdM, JvW and YV) were involved in the development of the ART model. Since the development of the ART model, the first author (LZ) has been performing research on the implementation and impact of the ART model, as part of her PhD. IdJ performs research on the High and Intensive Care model (HIC) and was involved in the analysis and reporting the results of this study to ensure objectivity.

### Ethical Considerations

Prior to participation, service users and significant others received information concerning the purpose and procedures of the study. At the start of the interviews, all participants provided informed consent. This study was approved by the Medical Ethical Committee of Amsterdam UMC, location VUmc.

## Results

In this section, the characteristics of the participants will be provided. Furthermore, we will present the results section according to the three central research questions of this study. We identified three themes regarding the Active principle, five themes related to the Recovery principle and three themes regarding the Triad principle of the ART model.

### Participant Characteristics

In total nineteen service users and five significant others (a partner, three mothers and one sister) from twelve different teams (of different organizations) participated in this study. 58% of the participants were male, four participants were younger than age 25, thirteen participants had the age between 25 and 50 and seven participants were older than 50 years old. We did not collect the diagnosis of the participants, as this was not deemed relevant for the aim of this study. Six teams did not recruit service users to participate for various reasons (e.g., service users not wanting to participate, being in crisis at the time of approaching, practical reasons such as shortage of time of care workers, or the team did not participate in the follow-up measurement at all).

### A: Active

Related to the principle ‘Active’ of the model, the following themes were established: service users being motivated by care workers, setting and working towards goals, and preferences regarding housing.

#### Being Motivated by Care Workers

Service users felt motivated by their care workers in various ways. They felt encouraged to be more active in difficult times:

Anyway, when you’re not feeling well, they motivate you to do things. At home, I easily just went to bed (- service user 3)

In addition, they felt motivated to engage in small tasks:

Service user: We are encouraged to do some tasks, such as putting the laundry in the washing machine. Preparing the table for dinner and stuff, I do that now too. So, I now perform some more tasks

Mother: Indeed, you are much more active, I agree. You also do things on your own. (- service user 10 and his mother)

Furthermore, service users felt stimulated to undertake leisure activities, for example because care workers reminded them of the daily activities that are organized every day, motivated them to engage in sports or take a walk, helped to awake timely for the daytime activities, or motivated them to start an education. One service user argued that it is important that care workers knew his personal interests, in order to be able to motivate him:

Motivation is difficult for me, it really depends, it has to be something I like otherwise you really can’t motivate me. I have the idea that they [the care workers] are getting to know me better, which of course helps in terms of interests and motivation (- service user 2)

#### Setting and Working Towards Goals

Most service users reported starting to set and work actively towards one or more goals. Several participants referred to care coordination meetings or treatment plan meetings to discuss these goals:

During a care coordination meeting, we examine what goals to work on. This takes place every six months […] Last time we discussed that I find it difficult to indicate the point at which I think, this is enough, for example at my work (- service user 16)

In addition, a sister of a service user preferred to be more involved in the process of determining goals, as she wanted to receive an overview of the personal recovery goals of her brother:

Yes, helping with personal goals would be something that could potentially be a little better, or we haven’t really received that information yet. It would really help when family knows what is being worked on, in some kind of overview (- sister of service user 7)

#### Preferences Regarding Housing

Many participants referred positively to the atmosphere on the location (ward/housing facility) during the interviews. They appreciated a homely atmosphere and a peaceful environment. A service user said:

I am actually quite happy with where I am now, I must say that the atmosphere here is just nice. […] it is a more normal homely atmosphere. It looks less like an institution (- service user 7)

Mostly older participants valued the location as a safe space. However, participants also mentioned they appreciated a personal space and having their own apartment or studio within the institution. They valued a quiet space which was often associated with a feeling of independency. Moreover, several service users preferred to live more independently, in a supported housing facility or to have their own home:

After this, I will try to get sheltered accommodation or my own home. You know, that I’m leaving. It’s not forever here (- service user 13)

Several service users mentioned the guided maximum length of stay at the location and their plans for the next step. Service users who talked about this in a positive way were often hopeful for the future. However, some limitations were mentioned by service users and significant others related to lengthy waiting lists for supported living or independent living. In addition, given the variability between the housing locations, for example in terms of the space and/or novelty of the building, some locations were perceived as a step backwards despite the increased level of independency.

### R: Recovery

We identified themes related to the four dimensions of recovery, namely recovery of health, recovery of identity, recovery of daily functioning, and recovery of community functioning. In addition, a fifth theme was developed regarding the preconditions for recovery-oriented care.

#### Recovery of Health

Regarding recovery of health, participants mentioned the attention to treatment and lifestyle. Concerning treatment, a service user appreciated the possibility for trauma therapy with EMDR, because she thought she ran out of options for her suffering. Care workers from the team who provide support also played a positive role in service users starting treatment:

Well, since September last year I started treatment and that took a few months to get motivated. They [the care workers] encouraged me; come on, now go start treatment, it will be good for you. Actually, they have never let go of that. When you come into care at [the current location], you have to start treatment. You can’t just only live at [the current location] (- service user 8)

Some service users were critical and did not agree with their medication or have a different opinion about their diagnosis:

Because there is a critical difference between how the clinicians perceive what happened to me and how the diagnosis is formulated as psychosis. But I see, I experienced it myself as a burnout. So, we differ in that, but I think it’s very nice that the difference is allowed to exist […] we can talk about this, we agreed that we differ in opinion (- service user 3)

A second topic related to recovery of health was lifestyle. Participants mentioned the attention for sports activities, losing weight and they enjoy cooking healthy food:

The cart [the traditional meal service] is fortunately gone, I thought that was really terrible. We have now started cooking ourselves at the ward. I really like the food now (- service user 15)

A mother saw her son obtaining a much healthier lifestyle:

I do see him becoming more independent. He used to be someone who hardly went outside, but he does now. He also looks much better now, much healthier. He sports every day. He’s doing an internship again and he likes it. Well, that’s also new because he used to do that too, but then he did it because we told him so, not because he wanted it himself. So, I see growth, yes (- mother of service user 10)

Last, a service user reported thinking about quitting smoking:

I smoke a lot, twenty-two cigarettes a day. I talk about quitting smoking with the doctor. Smoking is very unhealthy (- service user 7)

#### Recovery of Identity

Several service users appreciated the attention care workers had for their personal life story. One participant indicated the importance of this knowledge among care workers, in order to understand the person and provide tailored care and support. In addition, one service user also described that care workers helped him in his self-acceptance:

Yes, here I found out that I actually started to accept myself and understand who I am. […] My key worker helped me with this, I told him what happened and we talk about my thoughts. And I have a personal book in which I write down my thoughts (- service user 16)

#### Recovery of Daily Life

Several service users mentioned that care workers provided support in regaining grip on aspects of daily life and aiming for more self-reliance. First, financial support was mentioned several times by service users as well as by significant others. Most of the participants in this study had a financial guardian. Still, it remained important to have attention for a good management of finances:

I also have a financial guardian. They [the care workers] also provided information about how to deal with finances and what still had to be arranged. Now they help me to end the guardianship, and just pick up my own finances again with help of the care workers, so that I can do my finances myself again. (- service user 17)

Second, service users were supported to become more self-reliant in performing household tasks, including cleaning and groceries:

Sister: He has the most contact with her [key worker] because he also goes shopping together with a care worker and then [name key worker] basically always goes with him

Service user: But that has also changed, now I have to give receipts

Sister: “Oh, that’s changed again. The intention is that he will become more and more self-reliant. In the beginning, he did the shopping together with [name key worker]. Now it has changed again that he receives money in his account and then he has to hand in receipts. So now he has to go himself, [name key worker] goes no longer with him (- service user 7 and his sister)

Third, some service users receive support in personal care:

They [the care workers] provide structure to me, they help me to shower and with personal care as well (- service user 11)

#### Recovery of Community Functioning

Service users particularly valued the support regarding community functioning, as one service user said:

Yes, they help in multiple areas of my life. The fact that it is not only about your mental health, but also about the participation in the community and social aspect, that you are supported in this, I think they do it very well (- service user 11)

Service users and significant others mentioned that daytime activities played an important role in the recovery process. Service users undertook various daytime activities or started in small steps to work, according to their level of functioning. For example, they performed activities within the organization, such as gardening at the location, they helped at a local farm, or they worked in a restaurant. A service user mentioned:

Daytime activities are very important to me, so I am glad that the care workers also support me in this. Because it is very difficult for me to sit still, because otherwise I will suffer from psychosis. So yes, it’s great that they do that. […] I am helping out in a restaurant for a few hours a week (- service user 6)

Furthermore, service users undertake various hobbies and sports activities in the community, such as painting class, yoga or football. However, stigma plays a role here, as one service user said:

I just feel normal there [at the football club]. Nobody knows something about me, well my name, my age and that I played football before. But nothing about my mental health. I always have the idea that I would be treated differently when someone knows only just a little bit about this. No, I really like the environment where I am seen as a normal person (- service user 8)

#### Conditions for Recovery-Oriented Care

Service users and their significant others also mentioned factors influencing recovery-oriented care. Service users valued specific expertise in the team according to their needs and wishes. For example, one participant valued the expertise of addiction care in the team, since he struggled with an addiction. Another participant mentioned:

Well, I really think this is one of the best teams I’ve experienced. What is especially nice here, well, actually the different areas of expertise all fit together. […] One care worker is very good in relaxation and can really help you with learning to relax, relaxation exercises, a voice dialogue, those kind of things. The other one is very much focused on expertise by experience. Then you have the other person, […] she is very good at relationships and in the LGBTQI community (- service user 8)

The quote shows that this service user valued a diversity of expertise in the team. Another participant also mentioned the diversity in gender and ethnic background within the team. Additionally, service users appreciated that care workers act fast and adequately in case they are in need of more care or support, for example when they or other service users are in crisis. Communication between care workers was also frequently mentioned. Although some participants were positive about this communication, others reported that care workers were not always on the same page:

Sometimes with agreements, they are not on the same page. […] They sometimes think differently about a rule, and I find that annoying. So, when you have agreed on something with one person and the other does it slightly differently (- service user 11)

Service users and significant others were critical about staff turnover, including care workers in the team and treatment staff. A service user said:

I don’t understand why people leave and new people come every time. […] what do you mean shortage? Because every time there is a new one. Why do they have to leave, because every time they leave and the next day there is a new one. They can also just stay for ten, fifteen, twenty years. Or am I wrong to think this? (- service user 16)

### T: Triad

Themes related to the principle ‘Triad’ focused on the support from significant others, the contact with care workers and the contact with other service users.

#### Support from Significant Others

Most of the service users mentioned having frequent contact with one or more family members, friends or a partner. The significant others mentioned by service users in the interviews were: parents, siblings, children, grandmothers and in one case an aunt. Next to family members, one service user had regular contact with a group of friends from high school and one service user reported visiting a friend regularly for the weekend. In addition, two service users had a partner. In general, service users feel supported by contact with significant others. A service user said:

Yes, I receive much support from dad, my mother passed away. Dad knows me better than I know myself, from childhood of course (- service user 16)

In some cases, the contact was on a daily basis, through the phone and visits. In other cases, contact was sometimes difficult. One service user found it difficult to reach out when he struggled with symptoms of his mental illness. Also, due to the COVID-19 restrictions, the majority of the service users struggled with feelings of loneliness because they were not able to visit or receive visits from their significant others. In most cases, significant others played an active role in care and support. Sometimes significant others provided practical support:

[Name service user] is very neat and tidy with the room but then I just help a little with cleaning the floor and the bathroom. We do that together. (- mother of service user 10)

Besides helping in practical support, other roles of significant others that were mentioned were attending care coordination meetings, and involvement in setting personal recovery goals of service users and evaluating these goals. A mother felt heard during these meetings, when she was asked to give her opinion. Some participants reported that their family members did not attend these meetings, but received an update afterwards. It was also mentioned that some significant others only received an update when things went wrong.

However, several participants did not want to burden their significant others, or they were afraid that they worried too much about them. In addition, the partner of a service user found it difficult to understand what kind of role she played. She also wanted to provide support, but this was not explicitly discussed with her partner and care workers yet.

Three participants had no contact with family or friends at all. In addition, one service user did not want to re-establish contact with her family due to events in the past:

I don’t really have evaluation meetings, but I think that’s also because I don’t involve my network. […] But they [the care workers] are very much focused on this. […] it is indicated several times that they wanted me to involve my network, but that they also understand why I would rather not involve them. However, it is a recurring theme, yes. […] I understand the theory behind it. Also with ART and the triad of course, I’ve read a bit about it. […] So I, yes I do understand the theory behind it, just for some people it won’t work if you come from a vulnerable family or have a very, yes, particular background within your childhood and things like that, then I think it can also be counterproductive for people if such pressure is exerted on them (- service user 8)

This quote also illustrates that these care workers continued to talk about contact with family. More participants recognized that their care workers emphasized the importance of contact with significant others and that they also felt supported in this. In addition, the activities that were organized for family members and significant others were appreciated by service users as well as by significant others. At some locations, a ‘family day’ or a barbecue is organized yearly. Participants reflected that these are moments to connect with other family members in a low-key setting:

Well, for example, we sometimes have, that is not treatment, but we have had a family day. I really liked that. Then they invite my family to get acquainted with other family members in a different way. Then there is music and stuff, which I also like very much (- service user 18)

#### Contact with Care Professionals

Regarding contact with care professionals, service users appreciated friendliness, a positive approach and warmness:

When I’m sad or something. During the COVID times, they also gave me a hug (- service user 11)

Several values were deemed important by service users in contact with care professionals, namely honesty, involvement, respect, trust, equality and unconditional support:

They very much look at it this way; okay, why are you displaying this behavior and what could you do differently next time? They always say; we do not approve the behavior, but the person behind it, we just accept you. Because of that trust you can change, and an opportunity is offered to be able to change it (- service user 8)

Often participants mentioned the personal connection and one specific care worker who they preferred:

Mother: [name care worker] is very good

Service user: Yes, there is a care worker, [name care worker], and he is very understanding. He is not a peer worker, but he has suffered a lot himself and I think he is very good at putting himself in other people’s shoes. […] I think he, he has good… I don’t know how you call it…

Mother: Empathy

Service user: Empathy, yes (- service user 2 and his mother)

However, some service users reported having better contact with some care workers than with others. In addition, service users mentioned points of improvement regarding the reachability of the care professionals, including the difficulty to have contact in the evening and night and the value of knowing whom you talk to through the phone:

At the beginning, I sometimes called and then I got someone on the phone who I had never seen before. Yes, then it doesn’t feel comfortable to talk about everything and tell what’s going on. Or, if you’re texting the team, you have no idea who you’re talking to. Because it’s just the general phone number that I’m texting with. But who’s behind that… […] I have indicated a few times that I would rather like it if everyone could just be reached individually (- service user 17)

Contact with a key worker was frequently mentioned by service users and significant others. Most of the service users were positive and mentioned for instance that their key worker helped to reduce anxiety, ease concerns and helped to feel home:

My key worker helped me feel at home. […] He says that I can always turn to him, no matter what it is. I can always talk about anything, and I liked that. Because at the beginning I really had to get used to this place.(- service user 5)

Furthermore, service users valued that care workers were friendly to their family or partners and they trusted that when something happened, care workers would reach out to their significant others if needed. In addition, the importance of the support for significant others was reported. One partner felt taken seriously by the care workers:

Well, I really like the people. So, I get along really well with most of them, and they are very kind and involved. Really ask how things are going. […] And I can always text and call them. […] And they always respond to it, it is always addressed, taken seriously (partner of service user 17)

However, some significant others were also critical about the contact with care workers. A mother experienced too little contact with the care workers and key worker of her son. Additionally, a partner reported that the care workers were too late in contacting her:

It went somewhat wrong in the beginning, that they got me involved too late. So, I only came into contact with them when he was very deep in crisis. […] that is a point for improvement. Because of course it’s difficult when he’s in crisis and you don’t know anyone [of the care workers in the team] yet (- partner of service user 17)

#### Contact with Other Service Users

Most of the time contact with other service users on the ward or location was discussed in a positive light. First, they appreciated that they felt similarities with the other service users:

Yes, there are quite some people here who have about the same background or who really have the same roots as I have. (- service user 3)

Second, service users valued the possibility to seek contact with other service users when and if they felt like it:

But if I want contact, I can go to the living room because there are always people there. Talk about everything, different things. They do make jokes, very lame ones (- service user 7)

Or when they needed it, for example when they experienced loneliness:

It’s mainly because with me, it also has something to do with loneliness. I have a very limited social network and that’s why it’s nice for me here. There is always someone here, even though I withdraw very often. If you need it, it’s there (- service user 13)

Furthermore, during most of the interviews the joint activities were discussed, for example playing games, drinking coffee or cooking together. A last positive element of the contact between service users was that they were able to motivate each other. One service user explained how he started with working on a farm because another service user on the ward told him about this place, so they started to go together:

I had also indicated at the time that I wanted a daytime activity at [daytime activity location]. It is an open garden. And that’s where I started with two days a week and now three days a week. […] I help feeding the pigs. And I’m also going to learn how to drive with a bobcat, which is a kind of tractor. […] I didn’t want it at first, but because of a friend who also lives here, he does the same. So, he told me and then I had a care coordination meeting where I discussed it. So now we go there together (- service user 2)

In contrast, during one interview the contact with other service users was discussed in a negative way. This service user explained he did not appreciate the contact with the other service users:

We also have a group Whatsapp. I think everyone is in it now, but previously people weren’t in it because they didn’t get along. And as a result, there was often information that did not reach everyone. One time I had a fight about the laundry. I said in that Whatsapp group that I took out someone’s laundry, as it was my laundry day. But he didn’t read it. And then as payback, that person set my laundry at ninety degrees (- service user 10)

## Discussion

The aim of this study was to collect experiences and opinions from the perspective of service users and their significant others regarding care and support they received from teams that implemented recovery-oriented care with the ART model. We structured the interviews according to the main principles of ART, namely Active, Recovery and Triad. Service users and significant others recognized the three principles of the ART model and gave examples of important themes in their recovery processes that align with these principles. However, answers also indicated that despite team efforts, the three principles need some further consideration and were not always fully implemented according to the needs of service users and their significant others.

Under the principle, ‘Active’, service users reported to feel motivated in their recovery process, work actively on various personal recovery goals and have dreams for the future. Hope and optimism about the future are important factors in the personal recovery process, as also distinguished in the CHIME model (Leamy et al., 2011). A hopeful perspective and the belief that recovery is possible are important core values of the ART model (Zomer et al., 2020), which were confirmed from the perspective of service users and significant others in this study. In addition, regarding housing preferences, some service users valued the service as safe space, while others wanted to live more independently. This is consistent with the study of De Ruysscher et al. (2020), that found similar functions of a long-stay ward, including the promotion of independence and self-reliance but also a place where service users can ‘simply be’. This ambiguity could possibly lead to a tension between the promotion of living more independently in the community on the one hand or the focus on maintaining quality of life on the other hand. Regarding the promotion of living more independently in the community, there is the risk of demoralization when attempts fail (De Ruysscher et al., 2020; Moonen et al., 2016). In addition, previous research showed also the risk of social isolation, due to the difficulty of developing social relations with people in the community (Leff & Trieman, 2000). However, when merely focusing on maintaining quality of life, overall wellbeing and comfort in the current situation, the risk is that hope for the future fades away by accepting the status quo. A study of Trauer et al. (2001) found that housing preferences could change when asking people before or after they moved from long-stay wards to community care units. So, for every individual situation, the right balance must be found between cherishing the ward as a safe space and meeting the ambition for independence, based on the personal needs and wishes of service users over time. The age of service users might be a factor to take into account in this regard, as particularly older service users valued the service as safe space. Needs and wishes might also change when getting older (Zechner et al., 2019).

Regarding the second principle, ‘Recovery’, service users and significant others described experiences related to the different dimensions of recovery. First, recovery of health is, amongst others, promoted by addressing a healthy lifestyle, including healthy cooking and engaging in sports activities. This is in line with the increasing body of evidence regarding the effectiveness of lifestyle interventions for people with serious mental illness, also specifically for people in long-term mental health care, including a positive effect on somatic health (Looijmans et al., 2019), psychosocial functioning (Deenik et al., 2018a) and less use of medication (Deenik et al.,^2018^b). However, the question is whether in practice service users were offered such structured lifestyle interventions as described in these studies, as implementation still seems to be challenging (Deenik et al., 2019; Smit et al., 2022). Second, although mentioned by some participants, recovery of identity seems to be less recognized by service users and significant others. It can be argued that this might be more complex to address in care and support (Conneely et al., 2021) or there is relatively little attention devoted to interventions that specifically focus on self-identity (Van der Meer et al., 2021). Another explanation is that the four dimensions of recovery are intertwined and constantly interact with each other, thereby addressing recovery of identity already implicitly within the other dimensions. Third, it appeared that financial management is an important topic related to recovery of daily life for service users and significant others. It is well established that people with a serious mental illness have a poorer financial situation, which is of influence on personal and social wellbeing (Topor & Ljungqvist, 2017). More research needs to be performed on how to address financial issues properly in care and support, especially since most service users have a financial guardian. Also, based on this finding, it may be useful to include interventions regarding support in financial management in the ART model. Fourth, service users and significant others particularly appreciated care and support focusing on recovery of community functioning. This finding underlines the importance of building confidence in obtaining a role in the community, for example by community-based activities or supported to work towards employment, which is an important goal of mental health services and is also acknowledged in other studies (Burns-Lynch et al., 2016; Killaspy et al., 2019). However, taking (self)stigma into account in this confidence building is important, as also identified in the review of Chester et al. (2016).

The preconditions for recovery-oriented care that were mentioned by service users and significant others entail qualities of staff and composition of the team. Our findings are comparable with other qualitative studies that investigated experiences of service users regarding care and support (Horgan et al., 2021; Killaspy et al., 2019). The study of Horgan et al. (2021), like our study, underlined the diversity aspect, implying staff respecting diversity among service users. Our findings add that, according to service users, diversity should also be reflected within the team, including diversity in gender, ethnic background, but also a diversity in expertise within the team in order to meet the needs of service users in this regard. In addition, good communication among care workers in the team was also found in the study of Killaspy et al. (2019) to be helpful, but this was mainly pointed out by staff, not by service users.

Under the third principle of the ART model, ‘Triad’, the support from significant others, the contact with care workers and with other service users were discussed. First, most of the service users who were included in this study had good contact with one or more significant others. Our findings emphasize the need for contact with significant others regarding successes, and not only when things go wrong. However, insecurity prevails among significant others regarding the role they play and want to play in care and support. Additionally, not all service users wanted their significant others to be involved. In line with recently developed NICE guidelines, it is recommended to have open conversations with all parties regarding their wishes in this regard, as well as the expectations from all sides and with a regular interval (National Institute for Health and Care Excellence, 2020). It is also recommended to pay attention to this topic when there is no contact with significant others, as wishes and needs could change over time. Further research needs to be performed on interventions and tools to approach these situations in care and support. Second, we found that contact with care workers is of great importance. Much research has been performed on the importance of a good relationship between care workers and service users, without which recovery is not possible (Stanhope & Solomon, 2008). Earlier studies suggest that good contact with care workers is essential for contributing to improvements in quality of life, hope and community functioning (Roebuck et al., 2022). This involves a good working alliance, including the feeling of trust, being understood by care workers (Killaspy et al., 2019), being supported in personal recovery goals (Moran et al., 2017), and mirroring a positive sense of self (Bacha et al., 2020). In particular, several participants in our study referred to one specific care worker they valued the most. This finding leads to the question: are all care workers able to create a warm and trusting working alliance with service users? Proper education and reflection tools might contribute to this, as also recommended in the study of Bacha et al. (2020). Last, our findings indicate that service users and significant others value contact with other service users and perform activities together to reduce loneliness. The finding that this kind of contact can be supportive or beneficial in the recovery process was also reported by Killaspy et al. (2019). Our interviews provided a concrete example of this, including a service user who motivated his fellow service user to start daytime activities, when staff was not able to motivate him. The findings of this study provide a starting point to broaden the concept of ‘the triad’ in the ART model, not merely perceive this as an instrumental collaboration between service users, their significant others and care workers to work towards personal recovery goals, but also focus on the broad social network of service users, their preferences and wishes in this regard and stimulate the positive contact between service users at the location.

### Strengths and Limitations

This is the first study that provided insight into the perspectives of service users and significant others on care and support from teams working with the ART model. The qualitative methods that were used allowed us to create in-depth insight into their experiences and views. Some service users took part in the interview together with their significant other, which might have contributed to a safe space during the interviews. In addition, we were able to include service users and significant others from various locations throughout the Netherlands.

However, this study also has some limitations. First, care workers approached service users and significant others to participate in the interviews based on their willingness and ability to express their experiences properly. This might have resulted in a selection bias, as the most enthusiastic people might be included, leading to more positive findings, and participants needed to be able to concentrate for the interview. People with a severe mental illness may suffer from cognitive problems and have difficulties reflecting verbally on the questions of an interview(Green et al., 2000). We experienced that for some participants, communication through spoken word appeared to be challenging. Therefore, it might be worthwhile to investigate the use of other qualitative methods. For example, we can learn from the methods used in research including people with intellectual disabilities, such as shadowing (Van der Weele & Bredewold, 2021), the use of video (Kaley et al., 2019) or photo voice (Heffron et al., 2018; Sitvast et al., 2010). Second, only five significant others were included, of whom most were family members. Although every participating location was requested to approach significant others for the interview, it appeared to be difficult to include them. This limitation might reflect the lack of contact teams in long-term mental health care have with significant others in general. In addition, the interviews took place at the location of the service users, which might be a barrier for significant others to participate, as they needed to travel to the location to participate. Future research including more significant others, including family but also partners, friends and other people of importance to service users, might provide a more complete picture of the perspectives of significant others regarding care and support.

## Conclusion

This study provided insights into the experiences and views of service users and their significant others regarding care and support in teams working with the ART model. We found that participants of this study feel motivated in their recovery process, work actively on various personal recovery goals and have dreams for the future. Furthermore, service users and significant others reported how different dimensions of recovery are addressed in care and support they receive and mentioned specific conditions for recovery-oriented care. In addition, the support from significant others, contact with care workers, and contact among service users are deemed important. Based on the findings of this study, several recommendations for long-term mental health care can be made to improve care and support in practice. First, it is recommended to examine the individual situation to find the right balance between promoting to live more independently in the community and focusing on maintaining quality of life, based on the personal needs and wishes of service users and significant others. In addition, attention should be paid to the implementation of lifestyle interventions and interventions for financial management. It is also recommended to make recovery of identity more explicit in care and support and care workers should consider (self-)stigma in participation of service users in the community. Furthermore, it is important to have open conversations with all parties regarding their wishes and expectations on the collaboration in the triad. Contact with significant others should be an important topic of discussion, also when there is no contact, as whishes can change over time. Last, it is important to seek contact with significant others regarding successes and not only when things go wrong and care workers must positively stimulate the contact among service users. The findings of this study can also provide directions for the further development of the ART model.

## Data Availability

The data of this study are saved at the Amsterdam UMC and are accessible to members of the research team. For questions regarding the data collected in this study, please contact the first author (LZ).
